# Tailored Biliopancreatic Limb Length to 40% of Total Small Bowel Length in One Anastomosis Gastric Bypass (TABLE-40): Protocol of a Prospective Randomized Controlled Trial

**DOI:** 10.1007/s11695-025-08035-9

**Published:** 2025-07-10

**Authors:** Adam Abu-Abeid, Ronli Ovadya, Noa Gosher, Jonathan Benjamin Yuval, Anat Bendayan, Andrei Keidar, Shai Meron Eldar

**Affiliations:** 1 Tel Aviv Sourasky Medical Center, Tel Aviv, Israel; 2 Tel Aviv University, Tel Aviv, Israel

**Keywords:** Severe obesity, One anastomosis gastric bypass, Tailored biliopancreatic limb, Weight loss, Quality of Life

## Abstract

**Background:**

One anastomosis gastric bypass (OAGB) is the most common metabolic and bariatric surgery (MBS) in Israel, recognized for its effectiveness in achieving sustainable weight loss and mitigating obesity-related diseases. The metabolic outcomes of OAGB are significantly influenced by the length of the biliopancreatic limb (BPL). The objective of this study is to determine whether tailoring the BPL length to the total small bowel length (TSBL) results in more effective weight loss compared to patients undergoing OAGB with a fixed BPL of 180 cm. Efficacy and safety of this approach will also be evaluated, ensuring it does not lead to long-term morbidity or negatively impact patients’ quality of life.

**Methods:**

This multicenter, prospective, randomized trial will enroll at least 200 participants undergoing OAGB across three centers. Participants, aged 18 and older, will be randomized into two groups: one group will undergo a tailored BPL length based on TSBL (40%), while the control group will receive a standard BPL length of 180 cm. The primary endpoint is comparison of total weight loss after 1 and 3 years. Secondary endpoints include postoperative complications, quality of life, and improvements in obesity-related diseases.

**Results:**

The study will collect and analyze data as outlined in the protocol. The results will be reported upon completion of data collection and analysis.

**Conclusions:**

The TABLE-40 study will provide robust evidence on whether tailoring the BPL to 40% of TSBL offers higher weight loss outcomes compared to a fixed-length approach in OAGB. By assessing both efficacy and safety across multiple centers, the trial aims to inform best practices in limb length selection and optimize long-term outcomes for patients undergoing OAGB.

**Trial Registration:**

This study was registered at Clinicaltrials.gov (NCT06829381) on 29/1/2025.

**Graphical Abstract:**

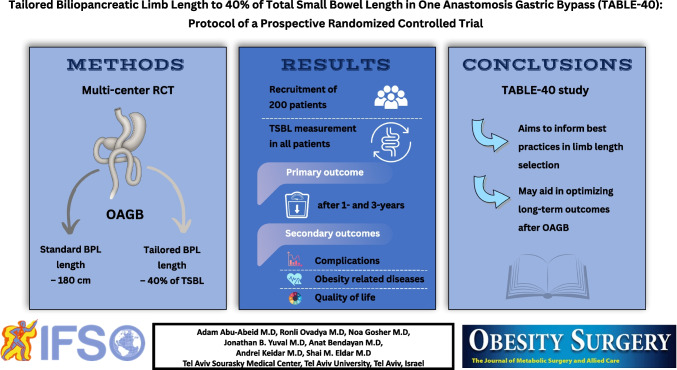

## Introduction

One anastomosis gastric bypass (OAGB) is reported to be the 3rd most commonly performed Metabolic and Bariatric Surgery (MBS) worldwide [1]. Its rapidly growing prevalence is supported by favorable literature showing OAGB to be an effective procedure with satisfactory outcomes in terms of sustained weight loss, improvement of obesity-related diseases, and relatively low rate of weight regain [2–4].

OAGB includes the creation of a relatively long gastric pouch providing a restrictive component, and performance of a single gastro-jejunal anastomosis, providing a hypo-absorptive component. Previous studies have shown that a biliopancreatic limb (BPL) length of 150–200 cm was both effective in weight loss outcomes and minimized the risk of malnutrition [5, 6]. The length of the BPL is a significant part of defining the metabolic outcomes after OAGB. Measurement of total small bowel length (TSBL) and tailoring of the BPL according to the TSBL is a controversial topic. There are reports recommending measuring the TSBL and ensuring at least 250–300 cm common channel (CC) to reduce the risk of malnutrition [5, 7]. TSBL can vary significantly in humans and ranges from 250 to 1300 cm [8].

In recent decades, there has been a shift towards personalized medicine, tailoring medical and surgical treatment to the individual characteristics of each patient [9, 10]. It is thought that by moving away from the “one size fits all” model, personalized medicine enhances efficacy, minimizes side effects, and improves patient outcomes. It is only natural to utilize the paradigm of personalized medicine in MBS and to tailor specific procedures to the patients’ morphometrics, such as TSBL.

The effect of different BPL lengths in OAGB has been reported—Felsenreich et al. conducted a review evaluating studies reporting various limb lengths used in gastric bypass procedures [11]. Their findings indicated that alterations in BPL length have a greater influence on the risk of nutritional deficiencies than on weight loss or remission of obesity-related diseases. Based on their analysis, they recommend constructing a BPL of 150–180 cm. In cases where a BPL exceeding 200 cm is considered, they advise measuring the TSBL to ensure that the CC remains adequately long.

The outcomes of patients after OAGB with a tailored BPL length are unclear. It is possible that these patients could have higher weight loss and metabolic outcomes. The purpose of this study is to evaluate if personalized tailoring of the BPL length to TSBL is effective and safe. Our primary outcome will investigate if tailored BPL is more effective in terms of percentage of total weight loss at 3 years compared to patients undergoing OAGB with a standard BPL length. We will also record perioperative and malnutrition-related outcomes in order to investigate the safety profile of the tailored BPL arm in comparison to the standard arm. We hypothesize that tailoring the BPL length to a percentage of the TSBL in patients undergoing OAGB will result in greater total weight loss after 1 and 3 years and result in comparable safety outcomes, including perioperative and malnutrition-related complications, compared to a fixed standard BPL length. We hereby present the protocol of the *T*ailored *B*iliopancreatic *L*imb *LE*ngth to 40% of TSBL study (TABLE-40 study).

## Methods

### Study Design and Patient Selection

This study is designed as a prospective, multi-centered randomized controlled trial to evaluate the impact of tailored BPL length compared to a standard BPL length in patients undergoing OAGB. Participants will be recruited from three or four centers, with potential participants identified during their pre-operative screening appointments.

Eligible participants will be patients aged 18 years and older and comply with MBS indications according to local Ministry of Health guidelines (a body mass index [BMI] ≥ 40 kg/m^2^ and BMI ≥ 35 kg/m^2^ with one or more obesity related disease). Preoperative evaluation and patient education in our study are conducted in accordance with standardized guidelines issued by the local Ministry of Health. All participating centers strictly adhere to these national protocols, ensuring consistency in preoperative care.

Exclusion criteria are shown in Table 1 and include special populations such as patients under 18 years of age, pregnancy during the study period, or those lacking judgment capacity, as well as individuals with previous bariatric surgery or TSBL < 450 cm, which is measured during OAGB. Patients with severe symptomatic gastro-esophageal reflux diseases and/or Grade C, Grade D, or Barrett’s esophagus, per endoscopy, are not considered candidates for OAGB.

**Table 1:** Exclusion criteria for patients in the TABLE-40 study

o < 18 years old
o Total small bowel length (TSBL) less than 450 cm (measured during surgery)
o Pregnancy during the study period
o History of previous bariatric surgery
o Clinically assessed cognitive impairment precluding informed consent

Informed consent will be obtained from all participants prior to enrollment, involving a detailed explanation of the study procedures, risks, and benefits, with ample time provided for consideration and questions.

Consent will be documented using a digital form via an institutional application, with a paper form available as a backup. Following consent, participants will be randomly assigned to either the tailored BPL group (40% of TSBL) or the standard BPL group (180 cm) using a computer-generated sequence to ensure balanced allocation. Block randomization will be employed to ensure balanced allocation between the study arms throughout the recruitment period. Variable block sizes will be used to minimize predictability and maintain allocation concealment. Patients are not blinded to treatment allocation as blinding was deemed impractical and potentially misleading in this context. Stratification will not be used in the randomization process, as our study population is expected to be relatively homogeneous with regard to key baseline characteristics, and randomization is anticipated to balance these across groups. While blinding of the surgical team is not feasible due to the nature of the intervention, data collection and analysis will be performed by researchers blinded to group assignment whenever possible.

### Intervention

#### Tailored BPL Group

During surgery, the surgeon will measure the TSBL. The BPL length will be determined as 40% of the TSBL. The rationale for this approach is to ensure a common channel length of at least 250–300 cm.

#### Standard BPL Group

During surgery, the surgeon will measure the TSBL. The BPL length will be measured and set to a fixed length of 180 cm.

#### Surgical Technique

All surgeries will be performed laparoscopic or robotic approach by MBS surgeons at the participating centers, adhering to standardized OAGB techniques. The dissection to create the gastric pouch begins below the level of the crow’s foot, and the pouch is created using a 40 Fr bougie for calibration, with the aim of constructing a pouch of at least 15 cm in length. The ligament of Treitz is identified and the TSBL is measured by a visual estimation. The anastomosis is performed in a side-to-side manner using a linear staple (30–40 mm) and the anastomotic opening is closed using a barbed suture. A routine blue dye leak test is performed. Mesenteric defects are not closed routinely.

### Postoperative Care

Patients are admitted postoperatively to the surgical ward and an Enhanced Recovery After Surgery (ERAS) protocol [12] is applied to all patients including early mobilization, fluid drinking at the evening of surgery, abstention of intravenous fluids and catheters. Patients are planned to be discharged on postoperative day 1. Criteria for patient discharge are shown on Table 2. All patients receive high-dose proton pump inhibitors (20 mg twice per day) for 3 months and continued venous thromboembolism prophylaxis with subcutaneous injection of enoxaparin 40 mg for three weeks. In addition, patients will be prescribed a standardized multivitamin supplement regimen as recommended by a clinical nutritionist, in accordance with established guidelines for postoperative care following MBS.

**Table 2:** Discharge criteria for patients after OAGB

• Patients ambulate freely with no assistance
• Postoperative pain controlled with non-opioid analgesia
• Vital signs within normal limits (pulse rate < 100/min; temperature < 37.5 °C; O_2_ saturation > 92%, mean blood pressure — 70–100 mmHg
• Ability to tolerate a minimum of 1500 mL of oral fluids within 24 h prior to discharge
• Absence of clinical indicators of postoperative complications
• Comprehensive understanding of post-discharge care and scheduled follow-up

### Data Collection

Data will be collected prospectively at baseline, intra-operatively, and during follow-up visits.

The following data points will be collected:

- Demographics: Age, gender
- Pre-operative: Weight, height, Body Mass Index (BMI), obesity-related conditions, previous abdominal surgeries, ASA score, Charlson comorbidity index (CCI), blood test results (hemoglobin, platelets, creatinine, albumin, vitamin D, B12, folic acid, iron, ferritin, transferrin, glucose, HbA1c, lipid profile, liver, thyroid and parathyroid function tests)
- Intra-operative: Date of surgery, duration of surgery, total small bowel length (cm), BPL length (cm), intra-operative complications.
- Post-operative: Post-operative complications (bleeding, leak, wound infection, VTE, surgical site infection), grading of complications according to Clavien-Dindo grading of complications [13], length of hospital stay, 90-day readmissions, 90-day reoperations.
- Follow-up (6 months, 1 year, 3 years): Weight, height, BMI, obesity-related conditions during follow-up, blood test results (as above), > 90-day complications, reoperations, quality of life measures will be measured according to the BAROS questionnaire [14]. Bowel habits and stool characteristics will be assessed according to the Fecal Score [15].

### Follow-Up

Participants will be followed up at 2 weeks, 3 months, 6 months, 12 months, and annually thereafter. At each visit, weight, obesity-related diseases, and any complications will be assessed. Blood tests will be performed at 3–6 month intervals to monitor nutritional status.

### Ethical Considerations

This study was approved by the Institutional Review Board (IRB) of Tel Aviv Sourasky Medical Center (Approval No. TLV-24-0586) on January 23, 2025. All data will be collected and stored in compliance with data privacy regulations. Participant data will be de-identified and stored securely on password-protected servers at the participating centers.

### Outcome Measures

#### Primary Outcome

The primary outcome is the difference in percentage of total weight loss (%TWL) between the two groups at 1 and 3 years postoperatively.

%TWL will be calculated as: (Pre-operative weight – Follow-up weight)/Pre-operative weight) × 100.

### Secondary Outcomes

- Difference in length of BPL and TSBL between the two groups.
- Changes in obesity-related diseases (diabetes, hypertension, dyslipidemia, sleep apnea) at 6 months and 1 year after OAGB. Remission of obesity-related diseases will be defined according to standard clinical criteria. In general, remission is considered when the patient is asymptomatic, no longer requires disease-specific medication, and relevant laboratory values are within normal limits. For example, type 2 diabetes remission will be defined as HbA1c < 6.5% without the use of antidiabetic medication, as per ADA criteria. Similar criteria will be applied for hypertension, dyslipidemia, and other obesity-related diseases, based on established clinical guidelines [16–18].
- Postoperative complications after OAGB—bile reflux, marginal ulcers, internal hernia, anastomotic strictures, and others—will be screened and compared.
- Incidence of nutritional complications (protein malnutrition, vitamin deficiencies) during the follow-up period.
- Changes in quality of life, as measured by the BAROS questionnaire at 6 months and 1 year post-surgery.

### Quality of Life Measures

#### MA-II Questionnaire

We will use the Moorehead-Ardelt Quality of Life Questionnaire II (MA-II), which is part of the Bariatric Analysis and Reporting Outcome System (BAROS), to assess quality of life at 6, 12 and 36 months postoperatively. The BAROS system was originally introduced in the 1990 s and was updated in 2009. The revised MA-II includes six questions evaluating self-esteem, physical activity, social life, work conditions, sexual activity, and the individual’s relationship with food. All six questions are equally weighted, with each item assigned a score of 0.5 points. A 10-point Likert-type scale is now utilized in the scoring system. As in the original BAROS questionnaire, the updated version maintains a weighted structure, with a central neutral value of 0 and positive or negative changes represented to the right and left, respectively.

Based on the final score, outcomes are categorized into five groups: failure (≤ 0), fair (0.1–1.5), good (1.6–3.0), very good (3.1–4.5), and excellent (4.6–6.0 points).

### Fecal Score (FS)

Altered bowel habits—such as loose stools, increased frequency of defecation, and even fecal incontinence—can significantly impact quality of life following bariatric surgery. To evaluate these changes, we are using the semi-quantitative Stool Frequency and Consistency Score, also known as the Fecal Score (FS), to assess bowel movement patterns at 6, 12, and 36 months postoperatively. The FS (Fecal Score) is calculated based on three components: fecal frequency, fecal consistency, and the impact on quality of life. Fecal frequency is assessed using a 5-point scale: less than twice per week (1 point), every two days (2 points), once daily (3 points), twice daily (4 points), and more than twice daily (5 points). Fecal consistency is also scored on a 5-point scale: watery stools (5 points), loose stools (4 points), normal stools (3 points), firm stools (2 points), and hard stools associated with constipation (1 point). The impact on quality of life due to frequent bowel movements is also rated on a 5-point scale, where 1 point indicates no impact, 2 points indicate mild impact such as anxiety related to bowel habits, 3 points indicate moderate impact including the use of aids (e.g., sanitary pads) due to occasional fecal leakage, 4 points indicate severe impact requiring restriction to locations near restrooms, and 5 points reflect very severe impact, including avoidance of leaving the house. The total FS ranges from 3 to 15, with higher scores indicating more frequent, looser stools and greater impact on daily functioning.

### Follow-Up Rate

Based on our previous experience in similar studies conducted at our center [19, 20], we anticipate a loss to follow-up rate of 10–20% after 3 years. Measures will be taken to minimize attrition, including scheduled follow-up reminders and coordination with clinical teams to ensure patient engagement throughout the study period.

### Statistical Analysis

#### Sample Size

Based on the previous literature of study cohorts which are similar to our expected study population [21–23], our hypothesis is that at 3 years the tailored group will have a 5% higher %TWL in comparison to the fixed group (40% vs. 35%). In order to detect a difference in %TWL at 3 years with a power of 0.8 and an alpha significance of < 0.05 and standard deviations of 12, 90 patients are needed in each arm for a total of 180 patients. Assuming a drop-out rate of 10%, enrollment of 198 patients is needed for sufficient power for the trial.

### Data Analysis

Statistical analysis will be performed using IBM SPSS Statistics software version 29. Continuous variables will be presented as means ± standard deviations, and categorical variables will be presented as frequencies and percentages. Baseline characteristics of the two groups will be compared using independent *t*-tests for continuous variables and chi-square tests for categorical variables.

The primary outcome (%TWL) will be compared between the two groups using an independent *t*-test. If data are not normally distributed, a Mann–Whitney *U* test will be used. Changes in obesity-related diseases and quality of life scores will be analyzed using paired *t*-tests or Wilcoxon signed-rank tests (as appropriate) to compare pre- and post-operative values within each group. Differences between groups will be assessed using independent *t*-tests or Mann–Whitney *U* tests. The incidence of complications will be compared between the two groups using chi-square tests or Fisher’s exact tests.

Multivariable regression analysis will be used to adjust for potential confounders (e.g., baseline BMI, comorbidities) in the analysis of the primary outcome. A *p*-value of < 0.05 will be considered statistically significant.

## Data Availability

No datasets were generated or analysed during the current study.
